# Mechanistic Framework for Establishment, Maintenance, and Alteration of Cell Polarity in Plants

**DOI:** 10.1100/2012/981658

**Published:** 2012-05-02

**Authors:** Pankaj Dhonukshe

**Affiliations:** Department of Biology, Utrecht University, Padualaan 8, 3584 CH Utrecht, The Netherlands

## Abstract

Cell polarity establishment, maintenance, and alteration are central to the developmental and response programs of nearly all organisms and are often implicated in abnormalities ranging from patterning defects to cancer. By residing at the distinct plasma membrane domains polar cargoes mark the identities of those domains, and execute localized functions. Polar cargoes are recruited to the specialized membrane domains by directional secretion and/or directional endocytic recycling. In plants, auxin efflux carrier PIN proteins display polar localizations in various cell types and play major roles in directional cell-to-cell transport of signaling molecule auxin that is vital for plant patterning and response programs. Recent advanced microscopy studies applied to single cells in intact plants reveal subcellular PIN dynamics. They uncover the PIN polarity generation mechanism and identified important roles of AGC kinases for polar PIN localization. AGC kinase family members PINOID, WAG1, and WAG2, belonging to the AGC-3 subclass predominantly influence the polar localization of PINs. The emerging mechanism for AGC-3 kinases action suggests that kinases phosphorylate PINs mainly at the plasma membrane after initial symmetric PIN secretion for eventual PIN internalization and PIN sorting into distinct ARF-GEF-regulated polar recycling pathways. Thus phosphorylation status directs PIN translocation to different cell sides. Based on these findings a mechanistic framework evolves that suggests existence of cell side-specific recycling pathways in plants and implicates AGC3 kinases for differential PIN recruitment among them for eventual PIN polarity establishment, maintenance, and alteration.

## 1. Introduction

When distribution of the subcellular structures and molecules generate asymmetry to mark one end of the cell distinct from the others, the resulting cellular asymmetry is called as “cell polarity.” Cell polarity is a universal feature of eukaryotic cells whereby distinct membrane domains are established and maintained to retrieve polar cargoes. The cell polarity marks are utilized for various functions such as cell side specifications, local outgrowth, cell cleavage plane orientation, and asymmetric cell divisions. Cell polarity thus drives differentiation, proliferation and morphogenesis of unicellular and multicellular organisms including yeast, animals, and plants. In unicellular organism such as yeast polarity is used largely for selecting the place of bud outgrowth. In multicellular organisms such as *Caenorhabditis elegans*, *Drosophila melanogaster*, and *Arabidopsis thaliana*, the main body axis is defined by the polarity of the single-celled zygote and this initial polar reference is followed by directional cell growth, oriented cell divisions, and spatiotemporal cell specifications leading towards an organized body patterning [1–7]. Consequently, abnormalities in cell polarity result in severe developmental defects that range from physiological imbalances, neurological degeneration, missing organs, malformed organs, and uncontrolled cell proliferation such as cancer [8, 9]. Therefore, cell polarity remains at the heart of development and represents one of the frontier areas of investigation in the field of cell and developmental biology.

## 2. PIN Proteins: Cell Polarity Compass that Navigates Plant Development

**Figure 1:**
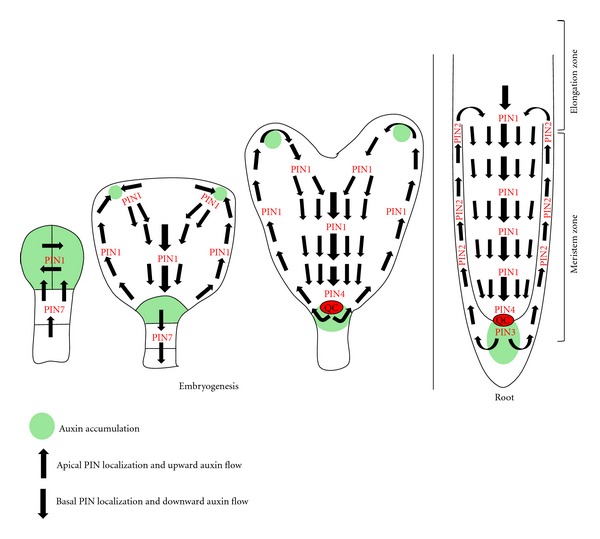
Polar-localized auxin efflux carrier PIN proteins direct auxin flow. Note that apical PINs guide upward auxin flow and the basal PINs guide downward auxin flow. Generation of directional auxin flux creates auxin accumulation zones that are required for proper progression of embryogenesis and for postembryonic organ functioning such as the root growth.

The intimate relationship between cell polarity and development is prominent in plants due to cell polarity-based plant embryogenesis and extensive postembryonic plant organogenesis. Plant cells do not possess polarity hallmark PAR proteins neither do they build membrane barrier tight junctions. Plant cells still display multiple polar domains and often alter their polarities in response to the developmental cues and to the environmental stimuli indicating an existence of flexible and plant-specific polarity mechanisms [10–14]. Fixed plant cell positions with respect to neighboring cells by the shared cell walls impose spatial constrains on generation, alteration, and utilization of cell polarity in plants. The analysis of cell polarity in plants is fueled by the discovery and analysis of plant-specific auxin efflux carrier PIN proteins which show polar localizations in various plant cell types in line with their roles in directional cell-to-cell auxin transport [11, 14–16]. PIN protein family comprises of 8 members, namely, PIN1, PIN2, PIN3, PIN4, PIN5, PIN6, PIN7, and PIN8 out of which PIN1, PIN2, PIN3, PIN4 and PIN7 display polar plasma membrane localizations [17]. In many auxin transporting polarized cells within the Arabidopsis embryo or within the Arabidopsis root PINs reside mainly at the apical (shootward) or basal (rootward) cell side (Figure 1), and, therefore, understanding mechanisms regulating apical or basal PIN localizations remains at the forefront of understanding plant cell polarity. PIN1, PIN4, and PIN7 play dominant roles during arabidopsis embryogenesis [18, 19]. Polarity of the zygote is elaborated by gradual polar localizations of auxin efflux carrier PINs. At the 2-cell embryo stage, PIN7 localizes to the apical side of the lower cell to evoke the transport of auxin towards the upper cell, and at 4-cell embryo stage, PIN1 appears at the facing sides (Figure 1) to equally distribute auxin in the proembryo [18]. Later on, PIN1 becomes polar in the protoderm (epidermis precursor) and in vasculature to trigger auxin flow towards the uppermost suspensor cell to convert it into hypophysis (Figure 1). Simultaneously PIN7 in the suspensor reverses its localization from the apical to the basal cell sides [18]. PIN1, PIN2, PIN4, and PIN7 remain largely at the basal cell sides in the root vasculature and in the root cortex [15, 18–20], whereas PIN2 remains at the apical cell side in the root epidermis (Figure 1) [19]. This shows that the same PIN is able to switch its localization in the same cell (e.g., the switch in PIN7 localization from the apical to the basal cell side in the suspensor during embryogenesis or the switch in PIN3 localization from symmetric to gravisensing cell side in columella during gravitropism) or in a different cell type (e.g., PIN2 localization in the epidermis is apical and PIN2 localization in the cortex is basal) based on the developmental response and cell type-specific inputs [18, 21, 22]. As this asymmetry in cellular PIN localization drives directional auxin fluxes, abnormalities in PIN localizations modify auxin status culminating into the range of auxin distribution defective phenotypes [23–33]. Because of this influence of PIN localization on the plant development via changes in the auxin distribution, mechanisms establishing, maintaining, and altering PIN polarity are of intense interest during the recent years.

## 3. Mechanistic Framework for Polar PIN Localization

**Figure 2:**
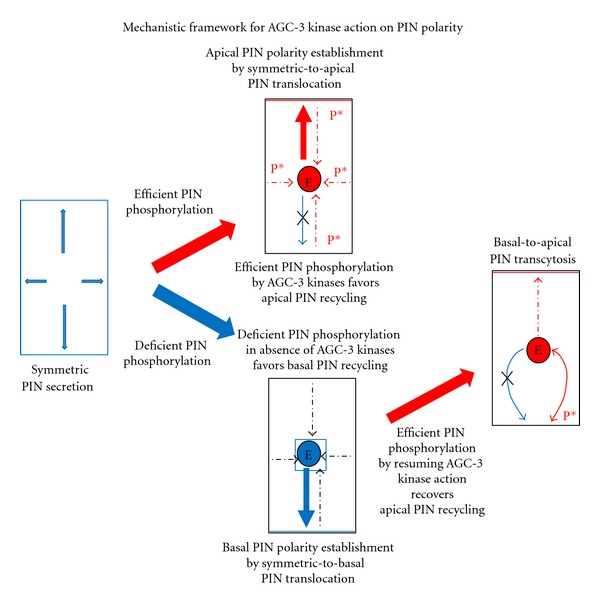
AGC-3 kinase-mediated phosphorylation status of PINs instructs directional PIN trafficking during establishment, maintenance, and alteration of PIN polarity. Note that polar plant cells possess cell-side-specific polar recycling pathways and phosphorylation-based sorting of polar cargoes recruits them in to a specific recycling pathway.

PINs are plasma membrane-resident transmembrane proteins that display the striking dynamics involving cycles of endocytosis and recycling [34, 35]. PINs utilize clathrin-mediated endocytic pathway for their internalization from the plasma membrane and an ARF-GEF-dependent recycling pathway(s) for their recruitment back to the plasma membrane [34, 35]. Ultrastructural studies and an impairment of clathrin pathway revealed that PINs predominately internalize through clathrin-mediated endocytic pathway [34]. An ARF-GEF GNOM was identified to be a PIN recycling regulator [32, 36, 37]. It was also shown that a drug Brefeldin A (BFA) which inactivates GNOM and thus impairs PIN1 recycling induces massive intracellular aggregation of PIN positive vesicles [32, 35, 36]. Photoconvertible tag-attached functional PIN versions revealed endocytic trafficking of PIN from the plasma membrane to the endosomes and its recycling from the endosomes back to the plasma membrane [34]. In addition, PINs display less lateral diffusion within plasma membrane in cell-wall-less and polarity-lacking protoplasts or in cell-wall-encased polar root cells indicating less lateral diffusion to be a context-independent integral property of PINs [24, 34, 38, 39]. Further, PINs reside in detergent-resistant membrane domains and interact with another plasma membrane located auxin transporter ABCB19, which stably associates with sterol/sphingolipid-enriched membrane fractions [40]. Genetic or chemical interference with cellulose synthesis impairs basal but not apical PIN1 localization, and drug treatment inducing retraction of plasma membrane from cell wall (called as plasmolysis) slightly enhances lateral PIN1diffusion [38]. Together, these findings show that PINs possess dynamic endocytic recycling and limited membrane diffusion property. Investigation of PIN polarity generation mechanism by real-time microscopy revealed that after synthesis PINs arrive at the plasma membrane symmetrically and then they internalize and recycle to the side of preference (Figure 2) [24]. When PIN internalization is impaired either by a short-term increment in auxin (impairing-clathrin dependent PIN endocytosis) [41, 42] or by an interference with the endocytosis regulator Rab5 [24, 43], PINs display largely symmetric localization suggesting an important role for endocytosis in PIN polarity generation [24]. The two step model of PIN polarity generation [24] brings PIN recycling regulator GNOM [32, 35, 36] into a new perspective by placing it at the second postendocytic step.

## 4. AGC-3 Kinase Action: Phosphorylation-Based Directional PIN Trafficking

Recent findings suggest that the AGC-3 kinase family members PINOID, WAG1, and WAG2 influence polar PIN localization in a cell type- and/or a context-dependent manner [23, 25, 30, 44, 45]. PINOID was identified as a regulator of polar auxin transport [46, 47], and later on it was shown to direct the PIN localization in two different tissues; the loss-of-function PID in the epidermis of shoot apical meristem results into basal localization of PIN1 instead of its normal apical localization in the WT and the gain of function PID in the root meristem results in apical localization of PIN1 instead of its normal basal localization in the WT [26]. Opposite polar localizations of PIN1 upon PID manipulation diverts auxin flux in opposite direction resulting into defects in aerial parts in the *pid* mutant and defects in root growth in the PID gain of function [26]. Recently, related WAG1 and WAG2 kinases were added to the PIN polarity modifier kinase toolkit showing that WAG1 and WAG2 bear the capacity to influence PIN polarity in manner similar to that of PID and in a PID-redundant manner [23]. Expression analyses of PID, WAG1 and WAG2 revealed that they all are largely expressed in the cell types where PIN (e.g., PIN2 in root epidermis) shows apical localization [23]. Further genetic, biochemical, and cell biology analyses identified the PID, WAG1, and WAG2 phosphorylation target residues present within the cytosolic loops of various PINs along with their importance for the polar PIN localization [23, 48]. Interestingly, PID, WAG1, and WAG2 phosphorylate PIN at the same residues suggesting conservation of phospho-target PIN residues [23]. Further, PIN localization pattern, PIN polarity-based polar auxin transport and auxin distribution-related plant phenotypes match for the kinase mutants and for the manipulation of phospho-deficient PIN versions in respective PIN mutants certifying the authenticity of kinase-targeted PIN phosphorylation residues [23]. After revealing the instructive role of kinases in regulating PIN polarity and identification of the PIN residues phosphorylated by them, the next task was to uncover the cellular mechanism by which PID, WAG1, and WAG2 regulate PIN polarity. Recent results pointed that all the three kinases localize at the plasma membrane in a largely nonpolar manner for impacting PIN polarization [23]. Detailed time lapse analysis revealed that after synthesis PID, WAG1, and WAG2 load at the plasma membrane in a membrane sterol composition-dependent manner to phosphorylate PINs [23]. This occurs when PINs arrive at the plasma membrane for the first time after their symmetric secretion or when PINs return to the plasma membrane after undergoing a cycle of endocytosis and recycling [23]. These results put forward a model whereby PID, WAG1, and WAG2 phosphorylate PINs largely at the plasma membrane after default nonpolar PIN secretion and thus provide a phosphorylation signature to the PIN [23]. This PIN modification is used for their postendocytic sorting at the intermediate compartments located along the trafficking route to eventually recruit PINs in differential recycling pathways for their deposition at the particular cell side [23] (Figure 2). It was also found that even with the conserved phosphorylation residues different PINs display different degree of sensitivity for their kinase-triggered polar localization [23]. This occurs in a PIN sequence- and cell-type-dependent manner suggesting that the sequence-based intrinsic differences within different PINs and the cell-type-based extrinsic differences both influence the PIN behavior [22, 23]. The gravity defects of *pin2* mutant line expressing PIN2 promoter-driven PIN1-GFP2 could be rescued by specific over expression of PID in epidermis indicating that localized PID manipulation possesses the capacity to locally alter PIN localization to resume and/or divert auxin flux [23]. Thus the identification of PIN phosphorylation residues along with the isolation of cellular mechanism for kinase action on PIN polarity now offers promising possibilities to manipulate PIN polarity in various contexts to further probe and utilize PIN polarity for modifying auxin transport-related plant developmental and response programs.

## 5. A Common Theme: Counteractions between Kinases and ARF-GEF Shift the Balance for PIN Relocation

Prolonged BFA treatment inhibiting GNOM function gradually shifts the basally localized PINs towards the apical cell sides via an intermediate BFA compartment stopover in a process termed as transcytosis [29]. This is considered to occur via basal PIN internalization and aggregation into BFA compartments (via a BFA-sensitive ARF-GEF such as GNOM) followed by BFA compartment to apical cell side PIN translocation (via a BFA resistant ARF-GEF) [29]. Interestingly, PID, WAG1, and WAG2 induction displays similar PIN translocations from basal to apical cell side with an intact GNOM functioning [23, 44]. Further analysis revealed that basal-to-apical translocation of PINs in BFA treatment-induced GNOM inactive situation depends on PID-, WAG1-, and WAG2-mediated PIN phosphorylation as phospho-deficient PINs (in *pidwag1wag2* triple mutant) are unable to achieve this [23, 44]. In addition, reduced phosphorylation of PINs by repressing kinase function leads to apical-to-basal translocation of PINs that presumably occurs via the GNOM-mediated basal PIN recycling. Thus the PIN phosphorylation status and the efficacy of ARF-GEF-regulated recycling determine whether PIN will translocate to apical or to basal cell side (Figure 2). Recent studies report utilization of conserved PID-GNOM (AGC-3 kinase and ARF-GEF) module for PIN polarity regulation during diverse developmental and response programs including phototropism [25], gravitropism [49], organogenesis such as lateral root formation [29], leaf epidermal cell indentations [30], and fruit valve margin formation [45].

## 6. Perspective

Even though the mechanism of PIN polarity generation from nonpolar secretion followed by PID-GNOM competition-based polar endocytic recycling seems to be solved [23, 24], there is yet much to learn about the ways plants cells establish, maintain, and alter PIN polarity. How PINs are destined to one particular cell side during the typical developmental or response program certainly remains a central question with a big challenge to solve the issue of directionality. With the availability of an exciting in vivo traceable tools such as photoconvertible fluorescent tag-attached functional PIN versions, the progressive and high-resolution microscopy platforms, constant identification of new PIN trafficking regulators, and acquisition of the finite details of the AGC-3 kinase and ARF-GEF PIN sorting module should bring us closer towards the answers.
